# Usability of Mobile Health Apps for Postoperative Care: Systematic Review

**DOI:** 10.2196/19099

**Published:** 2020-07-20

**Authors:** Ben Patel, Arron Thind

**Affiliations:** 1 Guy's and St Thomas' Hospital National Health Service Foundation Trust London United Kingdom; 2 East Surrey Hospital Redhill United Kingdom

**Keywords:** postoperative monitoring, postoperative care, mobile health app, telemedicine, smartphone, mobile phone

## Abstract

**Background:**

Mobile health (mHealth) apps are increasingly used postoperatively to monitor, educate, and rehabilitate. The usability of mHealth apps is critical to their implementation.

**Objective:**

This systematic review evaluates the (1) methodology of usability analyses, (2) domains of usability being assessed, and (3) results of usability analyses.

**Methods:**

The A Measurement Tool to Assess Systematic Reviews checklist was consulted. The Preferred Reporting Items for Systematic Reviews and Meta-Analyses reporting guideline was adhered to. Screening was undertaken by 2 independent reviewers. All included studies were assessed for risk of bias. Domains of usability were compared with the gold-standard mHealth App Usability Questionnaire (MAUQ).

**Results:**

A total of 33 of 720 identified studies were included for data extraction. Of the 5 included randomized controlled trials (RCTs), usability was never the primary end point. Methodology of usability analyses included interview (10/33), self-created questionnaire (18/33), and validated questionnaire (9/33). Of the 3 domains of usability proposed in the MAUQ, satisfaction was assessed in 28 of the 33 studies, system information arrangement was assessed in 11 of the 33 studies, and usefulness was assessed in 18 of the 33 studies. Usability of mHealth apps was above industry average, with median System Usability Scale scores ranging from 76 to 95 out of 100.

**Conclusions:**

Current analyses of mHealth app usability are substandard. RCTs are rare, and validated questionnaires are infrequently consulted. Of the 3 domains of usability, only satisfaction is regularly assessed. There is significant bias throughout the literature, particularly with regards to conflicts of interest. Future studies should adhere to the MAUQ to assess usability and improve the utility of mHealth apps.

## Introduction

Industry experts have forecasted significant growth in mobile app users [1]*.* Given this projected surge, mobile health (mHealth) apps offer a unique and readily accessible platform to the patient, surgeon, and innovator. mHealth apps are now being integrated into various sectors of health care, with over 318,000 [2] apps currently helping to track, educate, and diagnose [3].

One area of particular growth is the use of mHealth apps as a means of monitoring patients in the important postoperative period. Well-designed apps have the potential to encourage earlier discharge, reduce in-person follow-ups [4,5], rehabilitate [6]*,* aid clinicians in picking up surgical complications [7], and improve communication between patient and health care professional [8]. In addition to the economic and medical benefit of early discharge, postoperative monitoring apps have the potential to empower patients, giving them autonomy over their own health, which in turn might improve patient satisfaction and motivation for recovery [9].

The usability of mHealth apps is important [10,11] because those with poor usability will be less commonly used [12,13]. This is particularly significant in the postoperative period, given the focus of mHealth apps on rehabilitation, for which patient engagement is critical. One study revealed that around half of all mHealth app users stop engaging for various reasons, including loss of interest [14]. Despite this, little empirical research is undertaken to analyze the usability of mHealth apps before they are launched [15].

Several definitions and domains of usability have been previously defined without clear unification [11,16,17], but with several recurring themes. For example, the International Organization for Standardization (ISO) 3-pronged definition includes effectiveness (ie, whether users can use the product to complete their goals), efficiency (ie, the extent to which individuals expend resource in achieving their goals), and satisfaction [18]. Another definition [19] has been designed specifically for mHealth apps and includes factors such as mobility, connectivity, and additional cognitive load.

Different methods have been proposed for assessing domains of usability, such as the Post-Study System Usability Questionnaire [20] and the System Usability Scale (SUS) [21]. However, these tools were not originally created to evaluate mHealth apps. The Mobile App Rating Scale [22] was recently created for researchers and clinicians to assess the quality of mHealth apps, with the simpler user version of the Mobile App Rating Scale (uMARS) [23] being proposed shortly after. While quality of an mHealth app shares several components with usability, there are important differences.

Given the heterogeneity in definitions and methods used for assessing the usability of mHealth apps, one group has recently developed and validated the 21-item mHealth App Usability Questionnaire (MAUQ) [24]. This tool explores 3 domains of usability, which are in line with the ISO definition: (1) ease of use and satisfaction, akin to ISO satisfaction; (2) system information arrangement, akin to ISO efficiency; and (3) usefulness, akin to ISO effectiveness. This systematic literature review aims to determine whether the usability of postoperative mHealth apps is being rigorously assessed, using the validated MAUQ as the gold-standard reference. We consider which empirical methods are being used and analyze whether postoperative mHealth apps are indeed usable.

## Methods

### Database Search

The A Measurement Tool to Assess Systematic Reviews checklist [25] was analyzed before conducting this review, with all methodology being established prior to the review being conducted. A university librarian experienced in the field of systematic literature review methodology was consulted. The Preferred Reporting Items for Systematic Reviews and Meta-Analyses (PRISMA) [26] reporting guideline was adhered to for this review. Rayyan (Qatar Computing Research Institute) [27] software was used for the search.

Textbox 1 shows the questions that were defined.

The Medline, Embase, and Association for Computing Machinery Digital Library databases were searched. The search string was generated and aimed to provide maximum coverage while maintaining manageability. We defined 4 broad themes for our search. Terms within a theme were combined using Boolean operator OR, as seen in Table 1. Themes were then combined using Boolean operator AND.

**Search questions.**

1. Which dimensions of usability are dealt with most often?

2. Which empirical methods are used to evaluate usability?

3. In which surgical specialties are mobile health apps’ usability being evaluated?

4. What types of operating systems have been used?

5. What are the results obtained by the usability evaluation of the apps?

**Table 1:** Search strings for the 4 themes.

Theme	String
Mobile context	Smartphone OR smart phone OR mobile phone OR mobile device OR mHealth^a^ OR tablet
Software	App OR application OR operating system OR OS^b^ OR ios OR android OR windows OR google play
Postoperative	Postoperative OR post-operative OR surgery OR surgical OR operation OR perioperative OR peri operative
Usability	Usab* OR understandab* OR learnab* OR operab* OR attractive* OR user experience OR engag* OR satisf* OR adher* OR willing* OR accepta* OR effectiv* OR aesthetic OR intuitive*

^a^mHealth: mobile health.

^b^OS: operating system.

### Screening of Papers for Inclusion and Exclusion

Each study recruited from the initial search was evaluated to determine whether it should be admitted for analysis. The inclusion and exclusion criteria are shown in Textbox 2.

Screening of article titles and abstracts was performed by 2 authors independently. In situations where eligibility of a study could not be determined based on abstract alone, the full-text article was retrieved. We executed a full-text review of the remaining studies after title and abstract screening to further analyze appropriateness for inclusion. We analyzed all review articles to identify any other appropriate studies. We also reviewed the reference list of included papers.

**Inclusion and exclusion criteria.**

**Inclusion Criteria**

- The paper uses a mobile health app, defined as an application (rather than a web-based tool) on a portable device (including smartphones and tablets). We include apps designed both for the patient and for the health care professional. We include all types of apps, including monitoring, educational, and rehabilitation apps
- The paper analyzes the postoperative period, defined as the point at which the patient leaves the operating theater, having undergone a surgical procedure
- The paper studies usability of the mobile health app. Any level of assessment is included, from structured questionnaire to analysis of engagement or time spent on the app
- The paper must be a full paper (not an abstract)

**Exclusion Criteria**

- The paper is not written in English
- The paper was published before 2000, in keeping with the launch of the first smartphone, the Ericsson R380 (Ericsson Mobile Communications)
- The paper only uses web-based, text-based, or email-based technologies (no mobile health app). We want to concentrate on mobile health apps, given that they are the subject of such traction in the market
- The app is not targeted to the postoperative period. For example, surgical apps monitoring patients following trauma or burns are excluded if no operative intervention is used. Furthermore, nonsurgical papers (eg, monitoring patients with chronic pain) are excluded. In addition, apps only used for education of surgeons are excluded
- Inappropriate study types, including reviews, case reports, and feasibility/pilot studies without any real-life postoperative analysis
- App is not designed for humans

## Results

### Database Search Results

**Figure 1:**
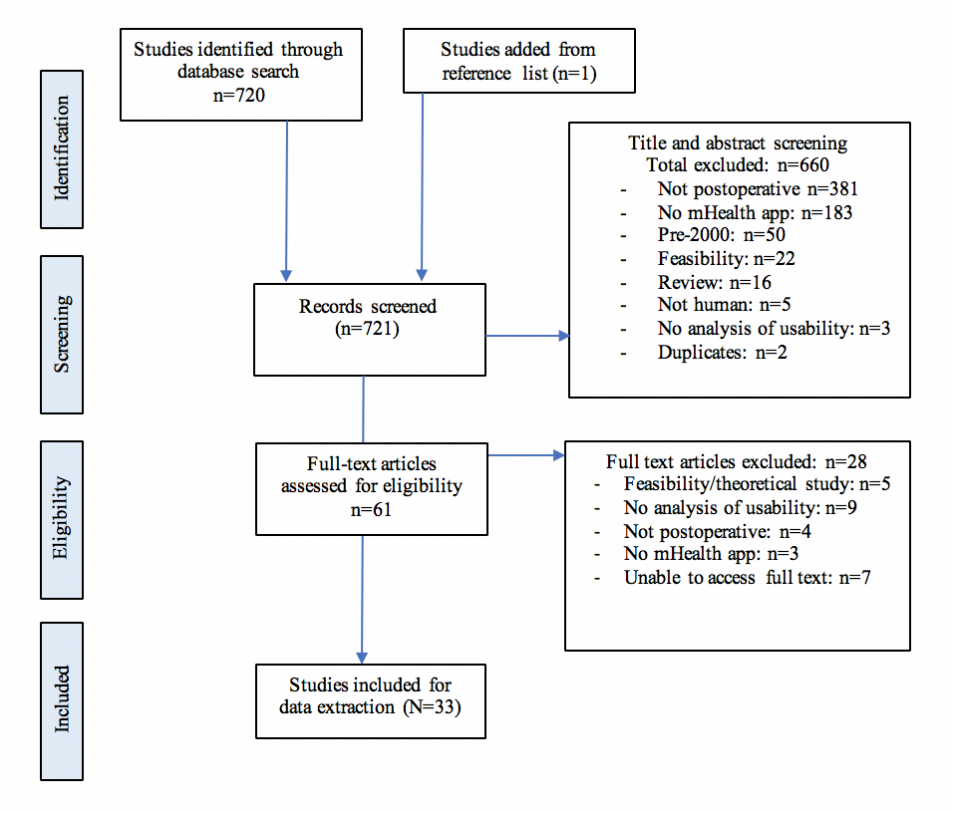
PRISMA summary of the literature search and exclusion process. mHealth: mobile health. PRISMA: Preferred Reporting Items for Systematic Reviews and Meta-Analyses.

The initial search and reference list screening identified 721 studies. After title and abstract screening, 660 were excluded, leaving 61 full-text studies to be assessed. Of these, 28 were excluded, leaving 33 studies included for data extraction. The PRISMA summary of the database search is presented in Figure 1.

### Study Characteristics

A total of 33 studies were included. Of the 33 studies, 21 were from North America (14 from the United States and 6 from Canada), 9 were from Europe, 2 were from Asia, and 1 was from South America. Most studies specified the type of mobile device used by participants. Smartphones were used in 22 studies, tablets were used in 9, smartwatches were used in 1, iPod touch (Apple Inc) devices were used in 2, and 3 studies did not specify. Regarding the operating system, 11 studies used iOS (Apple Inc), 5 used Android, 1 used Windows (Microsoft Corp), and 17 did not specify.

Among the included studies, mHealth apps were used within a wide range of surgical subspecialties, including orthopedics (8 studies), general surgery (6 studies), head and neck (4 studies), transplant (3 studies), pediatrics (2 studies), breast (1 study), vascular (1 study), neurosurgery (1 study), and others/multiple (7 studies).

Functionality was divided into 5 clear categories; 26 studies included monitoring of symptoms or wounds, 8 included educational content, 5 provided a communication platform, 5 included physiotherapy and rehabilitation, and 2 enabled medication management. App details are presented in Table 2.

Study characteristics are presented in Table 3. With regards to study design, 5 studies were randomized controlled trials (RCTs), 25 were prospective noncontrolled studies, and 3 were retrospective reviews. Sample sizes ranged from 4 to 494, with a median of 39 patients and a mean of 81 patients. Follow-up ranged from 30 minutes postoperation to 12 months postdischarge. The follow-up period was less than 7 days in 4 studies, between 1 week and 1 month in 15 studies, greater than 1 month in 9 studies, and not declared in 5 studies.

**Table 2:** App details, including the study, country of origin, type of mobile device used, app name, surgical subspecialty, and app function.

Study	Country	Primary mobile device (operating system)	App name	Surgical subspecialty	Function
Timmers et al [28]	Netherlands	Smartphone and tablet (—^a^)	Patient Journey App (Interactive Studios)	Orthopedics (elective total knee replacement)	Personalized educational information regarding pain; Physiotherapy; Wound monitoring; Self-care
Yadav et al [29]	India	Smartphone (—)	WhatsApp (Facebook Inc)	Endocrine surgery	Tele–follow-up including wound check and communication
Ramkumar et al [30]	United States	Smartphone (iOS)	TKR (Focus Ventures)	Orthopedics (elective total knee replacement)	Monitoring of mobility and range of movement using wearable sleeve; PROMs^b^; Analgesia need; Home exercise program compliance
Argent et al [31]	Ireland	Tablet (Android)	—	Orthopedics (elective total knee replacement)	Rehabilitation using an inertial measurement unit^c^, consisting of wearable sleeve; PROMs monitoring, including pain and perceived exercise difficulty
Brunner et al [32]	United States	Tablet (iOS)	Proloquo2Go (AssistiveWare)	Head and neck surgery	Augmentative and alternative communication in patients who are unable to speak postoperatively
van der Meij et al [33]	Netherlands	Smartphone (—)	—	Abdominal surgery (laparoscopic cholecystectomy, inguinal hernia surgery, laparoscopic adnexal surgery)	Information about surgical procedure; Insight into convalescence plan; Recovery monitor
Felbaum et al [34]	United States	Smartphone (—)	TrackMyRecovery	Neurosurgery	Postoperative instructions; Pain reporting; Wound monitoring
Goz et al [35]	United States	Smartphone (—)	—	Spine surgery	Postoperative communication through messaging app
Gunter et al [36]	United States	Smartphone (iOS)	WoundCheck	Vascular surgery	Wound monitoring using photographs and questionnaire
Gustavell et al [37]	Sweden	Smartphone and tablet (—)	Interaktor (Health Navigator)	Pancreatic surgery	Symptom monitoring; Education links to evidence-based care advice
Harder et al [38]	United Kingdom	Smartphone (iOS)	bWell	Breast surgery	Rehabilitation (arm exercises); Symptom monitoring
Higgins et al [39]	Canada	Smartphone (—)	QoC Health (QoC Health Inc)	Orthopedics (ACL^d^ reconstruction)	Symptom monitoring; QoR-9^e^ questionnaire
Highland et al [40]	United States	Smartphone (—)	mCare	Surgery using peripheral nerve block	Symptom control using DVPRS^f^
Khanwalkar et al [41]	United States	Smartphone (—)	HealthLoop	ENT^g^ (septoplasty and FESS^h^)	PROMs, including VAS^i^ pain score, PROMIS^j^, and SNOT-22^k^
Mata et al [42]	Canada	Tablet (iOS)	SeamlessMD (Seamless Mobile Health Inc)	Colorectal surgery	Milestones checklist; Symptom-monitoring questionnaires; Educational content
Nilsson et al [43]	Sweden	Smartphone (—)	Recovery Assessment by Phone Points	Day surgery	SwQoR^l^ questionnaire
Pecorelli et al [44]	Canada	Smartphone (—)	SeamlessMD	Colorectal surgery	Milestones checklist; Symptom-monitoring questionnaires; Educational content
Sousa and Turrini [45]	Brazil	Smartphone (iOS)	OrtogApp	Orthognathic surgery	Educational content; Communication platform
Sun et al [46]	Canada	iPod touch (iOS)	Panda (Balsamiq Solutions)	Pediatric surgery	Postoperative pain monitoring; Medication management
Tsapepas et al [47]	United States	Tablet (—)	Medication Regimen Education	Kidney transplant	Educational content
Scott et al [48]	United States	Smartphone (—)	SeamlessMD	Colorectal surgery	Symptom tracker; Photograph of wound; Temperature recording
Warren-Stomberg et al [49]	Sweden	Smartphone (iOS and Android)	Medipal (Novatelligence AB)	Day orthopedic surgery	Symptom questionnaire
Debono et al [50]	France	Smartphone and tablet (—)	—	Lumbar discectomy	Symptom monitoring
Gunter et al [51]	United States	— (iOS)	WoundCheck	Vascular and general surgery	Symptom monitoring; Photograph of wound
Ponce et al [52]	United States	— (iOS)	HelpLightning	Orthopedics and neurosurgery	Virtual examination
Jiang et al [53]	United States	Smartphone (Windows)	PocketPATH	Lung transplant	Data entry of health indicators; Self-monitoring
Chai et al [54]	South Korea	Tablet (iOS)	Self-Reporting Application	Thyroid surgery	Self-reporting of symptoms
Shellmer et al [55]	United States	— (Android)	Teen Pocket PATH	Solid organ transplant	Monitoring of medications
Sun et al [56]	Canada	Smartphone (—)	Panda	Pediatric surgery	Postoperative pain monitoring using electronic versions of FPS-R^m^ and CAS^n^
Jaensson et al [57]	Sweden	Smartphone (—)	Recovery Assessment by Phone Points	Day surgery	SwQoR questionnaire
Symer et al [58]	United States	Smartphone (iOS and Android) with paired smartwatch^o^	—	Colorectal surgery	Pain monitoring; Symptom monitoring; Patient reminders/alerts; Photograph of wound
Semple et al [59]	Canada	Smartphone or tablet (Android)	QoC Health	Breast and orthopedic surgery	Mobile version of the QoR-9 questionnaire
Bini and Mahajan [60]	United States	iPod touch (iOS)	CaptureProof	Orthopedic surgery	Physiotherapy videos

^a^Not available.

^b^PROMs: patient-reported outcome measures.

^c^Shimmer3; Shimmer.

^d^ACL: anterior cruciate ligament.

^e^QOR-9: quality of recovery 9.

^f^DVPRS: Defense and Veterans Pain Rating Scale.

^g^ENT: ear, nose, and throat.

^h^FESS: functional endoscopic sinus surgery.

^i^VAS: visual analog scale.

^j^PROMIS: Patient-Reported Outcomes Measurement Information System.

^k^SNOT-22: Sino-Nasal Outcome Test 22.

^l^SwQoR: Swedish Web Version of Quality of Life.

^m^FPS-R: Faces Pain Scale – Revised.

^n^CAS: color analog scale.

^o^Fitbit; Fitbit Inc.

**Table 3:** Study characteristics, including study design, number of patients included, duration of follow-up, method of usability analysis, usability domain, and selected usability results.

Study	Study design	Number of patients	Duration follow-up	Method of analysis of usability /outcome measure	Aspects of usability measured	Selected quantitative measure of usability
Timmers et al [28]	Multicenter RCT^a^	213	4 weeks	Measurement of patient usage; Interview of small group of patients (n=6)	Usefulness	App used 26 times/patient; Videos watched 36 times/patient; Qualitative reporting of usefulness
Yadav et al [29]	Prospective study (no control)	107	6 months	Self-created questionnaire	Satisfaction; Usefulness	1% unsatisfied across the questionnaire; 53% very satisfied with effectiveness; 78% very satisfied with app overall; Comfortable: 78% very satisfied; Convenience: 86%-91% very satisfied
Ramkumar et al [30]	Prospective study (no control)	22	3 months	Semi-structured interview	Satisfaction; Usefulness	A1: average score 2.6/10 (1=easiest to use; 10=most difficult)
Argent et al [31]	Mixed methods, including prospective study	15	2 weeks	Questionnaires (SUS^b^ and uMARS^c^); Semi-structured interview	Satisfaction; System information arrangement; Usefulness	uMARS average score 4.1/5 (SD 0.39); SUS average score 90.8 (SD 7.8)
Brunner et al [32]	Prospective preintervention and postintervention study	38	4 days	Self-created questionnaires; Measurement of usage	Satisfaction; Usefulness	66% used the app; 60% satisfied with the app; 85% felt it was helpful
van der Meij et al [33]	RCT	344	3 months	Measurement of usage; Self-created questionnaire; Semistructured interviews	Satisfaction	49.6% had used the app; Mean score for app 7.6/10
Felbaum et al [34]	Prospective study (no control)	56	—^d^	Self-created questionnaire	Usefulness	Usefulness ranged from 8.39-9.0 out of 10 (Likert scale)
Goz et al [35]	Prospective study (no control)	21	2 weeks	Measurement of usage/engagement; Self-created questionnaire	Satisfaction; Usefulness	82% satisfied (would recommend to others); 75% found useful (felt the app made it less likely for them to call the clinic); Engagement: 3.38 messages/person over 2 weeks
Gunter et al [36]	Prospective study (no control)	40	2 weeks	SUS (questionnaire); Measurement of usage	Satisfaction; System information arrangement	SUS average score of 87.2
Gustavell et al [37]	Prospective study (no control)	6	4 weeks	Measurement of usage; Semistructured interviews	Satisfaction; System information arrangement; Usefulness	Adherence to reporting daily was 84%; Other measurements qualitative
Harder et al [38]	Prospective study (no control)	4	8 weeks	Measurement of usage; Self-created questionnaire	Satisfaction; System information arrangement; Usefulness	Overall rating (Likert scale) 4.6/5; All used the app almost daily or several times/day
Higgins et al [39]	Retrospective case series	32	6 weeks	Interview; Self-created questionnaire	Satisfaction	Overall satisfaction was reported as excellent (43%), good (40%), fair (10%), poor (7%); 94% would use the app again
Highland et al [40]	RCT	24 (only 12 assessed usability)	10 days	SUS questionnaire; Additional questionnaire	Satisfaction; System information arrangement; Usefulness	SUS average score 76.26/100; No difference in convenience between intervention and standard of care (telephone follow-up)
Khanwalkar et al [41]	Prospective study (no control)	249	3 months	Measurement of usage	None	77.4% response rate (usage)
Mata et al [42]	RCT	50	4 weeks; Satisfaction measured at discharge	Measurement of usage; Self-created questionnaire using 4 items from S-CAHPS^e^	Satisfaction	Usage: postoperative day 0=94%, day 1=82%, day 2=72%, day 3=48%; 4/5 satisfaction across all 4 questions
Nilsson et al [43]	Prospective study (no control)	494	14 days	Measurement of usage (response rate)	None	Usage: day 1=86.8%, day 7=69%, day 14=57.5%
Pecorelli et al [44]	Prospective study (no control)	45	4 weeks	SUS questionnaire	Satisfaction; System information arrangement	SUS average score 87/100
Sousa and Turrini [45]	Prospective study (no control)	30	—	SUS questionnaire; Satisfaction measured according to experience sampling method technique; Usage	Satisfaction; System information arrangement	SUS average score 79.8/100, 73.3% >68 (cutoff), 100% >50 (acceptable); Satisfaction 82.9%; Usage: 100% used at least once, 40% used 2-3 times, 10% used 5 times, 20% used >5 times
Sun et al [46]	Prospective study (no control)	29	—	CSUQ^f^ Unstructured interviews	Satisfaction; System information arrangement; Usefulness	Median CSUQ score 2 (IQR^g^ 1-3); 93% found app easy to use; 59% would use the app at home
Tsapepas et al [47]	Retrospective study	282	—	Self-created questionnaire	Satisfaction	Satisfaction rated 4 or 5 in 92%
Scott et al [48]	Prospective study (no control)	20	14 days	SUS questionnaire; Semi-structured interview; Measurement of usage	Satisfaction; System information arrangement; Usefulness	Median SUS 95/100; Usage: 30% did not use after discharge
Warren-Stomberg et al [49]	Prospective study (no control)	101	1 week	Measurement of usage	None	55/101 used the app; Of those that used the app, 53% used >13 times out of possible 15
Debono et al [50]	Prospective study (no control)	60	15 days	Telephone interview	Satisfaction; Usefulness	1 (poor) to 4 (excellent) scale: Overall satisfaction 3.4 Usability 3.5 Usefulness at home 3.2 Facilitating return at home 3.1; 91.6% would use the device again
Gunter et al [51]	Prospective study (no control)	9	—	SUS questionnaire	Satisfaction; System information arrangement	Average SUS score 83.3/100; 55.6% were able to complete the tasks independently
Ponce et al [52]	Prospective	31	24 days	15-point questionnaire	Satisfaction; Usefulness	Reassurance 4.6-4.8/5; Useful 4.5-4.8/5; Satisfaction 4.2-4.6/5
Jiang et al [53]	Secondary retrospective analysis of previous RCT data	96	12 months	Technology acceptance subscales used to measure: intention to use (1 item); perceived usefulness (4 items); and perceived ease of use (4 items)	Satisfaction; Usefulness	85% strongly agree with intention to use item; 80% gave high rating of perceived usefulness (>24/28); 82% gave high rating of perceived ease of use (>24/28)
Chai et al [54]	Prospective comparison study (nonrandomized)	54	14 days	Self-created questionnaire	Satisfaction; Usefulness	Satisfaction was >7.2/10 across all 4 items on questionnaire
Shellmer et al [55]	Prospective study	7	6 weeks	8/16 questions from PSSUQ^h^ survey	Satisfaction; System information arrangement; Usefulness	Satisfaction 1/7 (1=strongly agree); Ease of use 1/7; Felt comfortable using application 1/7; “I could clearly tell when I missed my medication” 1/7; Liked tracking medications 3/7; Helpful to track medications 2/7
Sun et al [56]	Prospective study	66	30 minutes postoperation	Single question asked regarding preference of monitoring (app vs paper version of questionnaire)	Satisfaction	76%-81% preferred the app over the paper version
Jaensson et al [57]	Prospective study	10	—	Self-created questionnaire on system layout and technical issues, satisfaction, and usefulness	Satisfaction; System information arrangement; Usefulness	—
Symer et al [58]	Prospective study	31	30 days	Measurement of usage; Self-created questionnaire	Satisfaction; System information arrangement; Usefulness	83.9% used the app 70% of the time; 89.3%: easy to navigate; 88.9%: easy to use; 85.2%: survey questions relevant for identifying problems related to readmission; 66.7% found reminders useful; 92.9% would recommend to others
Semple et al [59]	Prospective study	65	30 days	Self-created survey; Interview; Usage	Satisfaction	Satisfaction 3.7-3.9/4; 100% wiling to use in future; 100% surgeons found platform intuitive and easy to use; Usage: mean number of logins 19.3-23.9/30 days; Mean number of photographs uploaded 38-63/30 days
Bini and Mahajan [60]	RCT	29	24 weeks	Self-created survey; Free-form feedback; Usage	Satisfaction	Ease of use: 3.9-4.4/5; Satisfaction 4.2/5

^a^RCT: randomized controlled trial.

^b^SUS: System Usability Scale.

^c^uMARS: user version of the Mobile App Rating Scale.

^d^Not available.

^e^S-CAHPS: Surgical Care Consumer Assessment of Healthcare Providers and Systems.

^f^CSUQ: Computer System Usability Questionnaire.

^g^IQR: interquartile range.

^h^PSSUQ: Post-Study System Usability Questionnaire.

### Usability Analysis

Regarding the method of usability analysis, usage (ie, monitoring of user engagement with the app) was used in 15 studies and was the only usability analysis employed in 4 studies. Interviews were used in 10 studies. Self-created questionnaires were used in 18 studies. Validated questionnaires were used in 9 studies. Of these, 7 used the SUS questionnaire, 1 used the uMARS questionnaire, 1 used the technology acceptance subscale, and 1 used the Computer System Usability Questionnaire (CSUQ).

We have categorized the domains of usability according to the MAUQ. A total of 28 studies covered ease of use and satisfaction, 11 studies covered system information arrangement, and 18 studies covered usefulness.

Average SUS scores ranged from 76 to 95 out of 100, with a median score of 87. The uMARS score was 4.1 out of 5. The CSUQ score was 2 out of 7 (whereby a score of 1 would indicate greatest usability).

### Bias

There is significant potential for bias in studies evaluating the usability of mHealth apps. Hidden agenda bias and secondary gains bias were common and seemingly underreported in the literature. Of the 33 included studies, 8 officially reported authors’ conflicts of interest, stating that they held shares in the app. Furthermore, several of the study groups were provided with the apps free of charge [28], which has clear implications on the usability domain of satisfaction; users who have paid for an app might be expected to have higher expectations than those who have been given an app for free. Perhaps more worryingly, a number of groups [38] declared no conflict of interest, despite seemingly being founders of their app.

Nonresponse bias is a further concern. Some studies, such as Pecorelli et al [44], had high response rates (96%) to usability analyses. However, others, such as Nilsson et al [43], had much lower rates (57.5% on day 14), and some [51] did not disclose the proportion of responders. Nonresponders to usability analyses are more likely to have reported poor usability. Therefore, studies with high rates of nonresponders are likely to have inflated usability results.

Population bias is a further issue. Younger audiences are likely to be more adept at using mobile technologies. Therefore, studies that include a younger demographic are likely to demonstrate inflated usability results. Additionally, the generalizability of results from studies [44] that included patients that were not used to mobile technologies may be limited and may change in the future, when greater numbers of older patients are used to mobile technologies.

## Discussion

### Principal Findings

To our knowledge, this is the first comprehensive systematic review to assess usability of mHealth apps in postoperative management. This review identified 33 studies evaluating the usability of mHealth apps in the postoperative period across a broad range of surgical subspecialties, demonstrating the growing interest in this area. Most of the included studies were derived from the United States and Europe, which appear to be hubs of innovation in the field. Unsurprisingly, smartphones were the most commonly used devices. However, we suspect that wearable devices such as smartwatches, which have additional monitoring capabilities such as electrocardiogram monitors, will play an increasingly important role in the future [61].

With respect to study designs, 25 of 33 studies were prospective noncontrolled trials. There were 5 RCTs, but usability was never a primary end point in these studies. We feel RCTs comparing mHealth apps to normal practice (eg, in-person follow-up, telephone follow-up, or no follow-up) would be particularly beneficial in assessing the domains of satisfaction and usefulness. It has also been suggested that mHealth app interventions are associated with a falsely heightened level of user satisfaction due to patients’ affinities for their digital devices [62]. This could be minimized by comparing postoperative mHealth apps to a sham app. However, we also acknowledge that RCTs have previously been described as an impractical evaluation methodology for mHealth apps, due to their prolonged duration from recruitment to results and their high costs [63].

The methodology for assessing usability was generally poor. The majority of analyses used simplistic self-created questionnaires that asked rudimentary questions focusing on the domain of satisfaction (28/33 studies) rather than other domains of usability. Indeed, only 11 of the 33 usability analyses assessed the domain of system information arrangement. We would argue that formal usability analyses should cover all 3 common domains of (1) satisfaction, (2) usefulness, and (3) system arrangement, according to the ISO definition of usability [18]. Validated questionnaires are helpful in assessing these areas reliably. Only 9 of the 33 included studies used validated questionnaires, most of which used the SUS. The SUS is a Likert scale made up of 10 questions. The average SUS score is 68 out of 100, meaning that all 7 studies that used the SUS scored above average in terms of usability. Although the SUS is a quick and cheap means of assessing usability, it was created in 1986, before the first smartphone or the concept of an app was realized. The SUS has not been validated for assessing mHealth apps. In comparison, the MAUQ was recently proposed and validated for use in mHealth apps in a population of English-speaking adults [64]. This is the gold-standard reference for analysis of mHealth app usability. While scores on the MAUQ have previously been shown to correlate with the SUS, this is not a strong correlation (*r*=0.643), thereby highlighting the inadequacy of studies that have only used the SUS.

A major concern in these studies is the risk of bias. A number of the studies’ authors have a financial interest in the usability of their apps, with high user satisfaction making adoption by hospitals and investors more likely. Furthermore, devices were sometimes provided free of charge, which could influence the feedback from users.

### Conclusions

mHealth apps have significant potential during the postoperative period for encouraging earlier discharge, improving patient engagement, and offering a safety net for early identification of complications. Thorough analysis of usability is critical to the adoption of these novel technologies in the postoperative period; those with poor usability will have little impact in health care. According to this review, usability analyses to date have been substandard. They have focused on satisfaction, a narrow dimension of usability, with simplistic self-created questionnaires. Furthermore, there is a significant risk of bias, given the common conflicts of interest among authors of published studies. We hope this review changes future practice, with researchers undertaking more robust assessments of usability by employing validated questionnaires, such as the MAUQ, in blinded RCTs.

## Abbreviations

CSUQ: Computer System Usability Questionnaire
ISO: Organization for Standardization
MAUQ: mHealth App Usability Questionnaire
mHealth: mobile health
PRISMA: Preferred Reporting Items for Systematic Reviews and Meta-Analyses
SUS: System Usability Scale
uMARS: user version of the Mobile App Rating Scale
